# Metabolic Demand and Muscle Activation during Different Forms of Bodyweight Supported Locomotion in Men with Incomplete SCI

**DOI:** 10.1155/2014/632765

**Published:** 2014-05-21

**Authors:** Alyssa M. Fenuta, Audrey L. Hicks

**Affiliations:** Department of Kinesiology, McMaster University, 1280 Main Street West, Hamilton, ON, Canada L8S 4L8

## Abstract

Body weight supported locomotor training uses neuroplasticity principles to improve recovery following a spinal cord injury (SCI). Steady state locomotion using the same body weight support (BWS) percent was compared in 7 males (42.6 ± 4.29 years) with incomplete SCI and matched (gender, age) noninjured controls (42.7 ± 5.4 years) using the Lokomat, Manual Treadmill, and ZeroG. The VO2000, Polar Heart Rate (HR) Monitor, and lower limb electromyography (EMG) electrodes were worn during the 2-minute sessions. Oxygen uptake (VO_2_) and HR were expressed as percentage of peak values obtained using progressive arm ergometry; VO_2_ was also expressed relative to resting metabolic equivalents (METS). Filtered EMG signals from tibialis anterior (TA), rectus femoris (RF), biceps femoris (BF), and medial gastrocnemius (MG) were normalized to ZeroG stepping. The Lokomat required 30% of VO_2_ peak (2METS) compared to ~54% (3METS) for Manual Treadmill and ZeroG sessions. HR was 67% of peak during Lokomat sessions compared to ~83% for Manual Treadmill and ZeroG. Muscle activation was higher in treadmill conditions compared to the ZeroG primarily due to increased BF activity. At the same level of BWS, locomotion using the Manual Treadmill or the ZeroG is more aerobically demanding than the Lokomat. Treadmill modalities encourage greater hip extensor activation compared to overground locomotion.

## 1. Introduction

**Figure 1:**
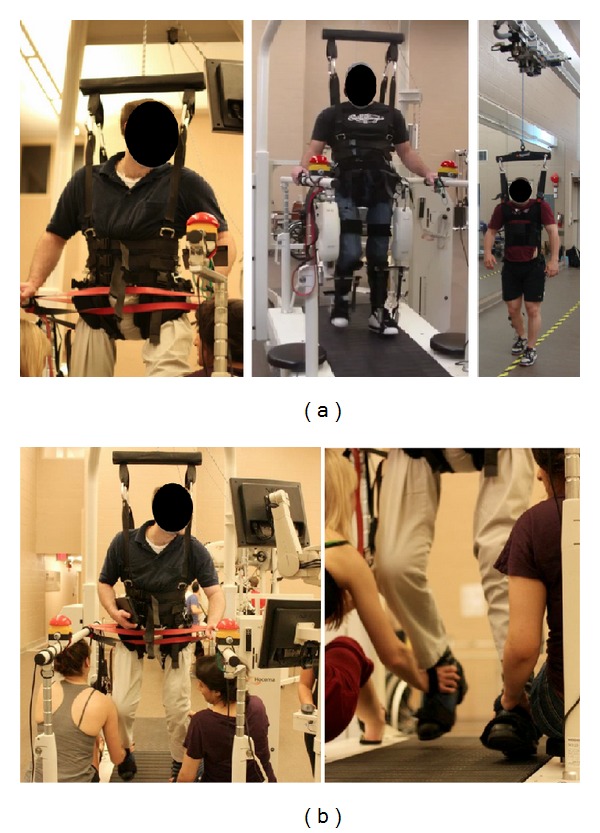
(a) Body weight supported training devices used during walking sessions; from left to right: Manual Treadmill, Lokomat, and ZeroG. All devices provided body weight support (BWS) through a harness. The Andago GmbH treadmill (Loko) was used for robotic assisted (Lokomat) and unassisted (Manual Treadmill) walking sessions. The ZeroG had a custom series elastic actuator that traveled along an overhead trolley, which provided dynamic BWS while individuals performed overground walking sessions. (b) Manual Treadmill sessions normally completed with therapist assistance (not provided in this study) at both legs. The Andago GmbH treadmill is used during these sessions without the robotic orthosis providing individuals with greater degrees of freedom during training.

Body weight supported locomotor training (e.g., treadmill) is a modern approach to spinal cord injury (SCI) rehabilitation, which provides an interactive, task-specific training environment that is believed to promote “rewiring” of synaptic connections [1–3]. The amount of loading experienced by an individual is manipulated in an attempt to compensate for uncontrolled spinal reflexes and motor losses associated with the injury. In doing so, these interventions offer the possibility of including gait retraining earlier in rehabilitation programming. Research suggests that the eventual transfer to unsupported overground walking is limited to those with incomplete SCI (American Spinal Injury Association Scale (AIS) C-D) as they benefit from retained brain-body connections, particularly with the cerebellum [4]. Presently, there are a variety of training devices available for individuals to consider using during rehabilitation (Figure 1). Alexeeva and colleagues [5] suggest that an effective intervention to improve locomotor ability in this subpopulation is overground locomotor training with 30% body weight support (BWS) that takes place 1 hour per day, 3 times per week, for 10 weeks in duration. It is important to note, however, that* optimal* training strategies to enhance locomotor function have yet to be established for individuals with incomplete SCI.

Locomotion targets the lower limbs and has an associated metabolic cost, which can be reduced by unloading an individual through the use of a harness. Following treadmill training, normalization of the gait pattern is possible allowing individuals to become more efficient at completing the task of walking [6, 7]. In fact, there is evidence to suggest metabolic cost decreases by as much as 68% following training in individuals with SCI [8]. To our knowledge, only one study [9] has* simultaneously* collected metabolic and EMG measurements during robotic and therapist assisted treadmill locomotion in individuals with incomplete SCI. These researchers determined that* voluntary* effort is required during robotic-assisted locomotion to achieve metabolic costs and hip flexor activity similar to those associated with therapist-assisted locomotion. It was suggested that therapist-assisted treadmill training programs should be used as progressions from robotic-assisted sessions.

The purpose of this study was to investigate oxygen demand and muscle activation patterns at the* same* level of BWS while individuals with incomplete SCI completed a locomotor training session using (a) the treadmill with robotic assistance (Lokomat), (b) the treadmill* without* therapist assistance (Manual Treadmill), and (c) an overground training system (ZeroG). It was hypothesized that the highest levels of oxygen uptake and muscle activation would occur during locomotion using the ZeroG and this would be higher in persons with incomplete SCI versus gender and age matched able-bodied adults.

## 2. Methods

### 2.1. Participants

Adults (18–65 years of age) with SCI were eligible to participate if their injury was (a) chronic (1 + year) or (b) incomplete (AIS C-D, sensory and/or motor function at S4/5) and (c) if they could comfortably complete walking sessions using the Andago GmbH treadmill (Loko, Germany) with manual and robotic (LokomatPro, Hocoma, Switzerland) assistance, as well as overground using the ZeroG (ZeroG; Aretech, LLC, Ashburn, VA). Each participant with a SCI was gender and age (±5 years) matched to an able-bodied adult, who served as the control group. For the ZeroG session, participants were required to be able to walk with less than or equal to 68 kg (150 lbs) offset, as this was the maximum amount of dynamic BWS (constant rope tension) the machine could provide. Participants did not consume any food (except water) in the four hours preceding testing and did not consume caffeine or alcohol or participate in strenuous exercise in the 24 hours prior. Familiarization with the laboratory environment, equipment, and test procedures occurred during prescreening. Written informed consent was obtained from all participants with a protocol approved by the McMaster Research Ethics Board.

#### 2.1.1. Baseline Assessments

At the first session, demographic and anthropometric measures of height (cm) and weight (kg) were recorded. Lower length measurements were taken to properly fit individuals to the Lokomat and individuals were fitted to harnesses for the respective devices. The amount of BWS to be used for all walking sessions was determined to be the percentage of body weight offset that allowed individuals to take steps using the ZeroG without excessive knee flexion.

Participants performed a VO_2_ peak test to volitional fatigue on an electronically braked arm ergometer (Angio V2; Lode BV, Groningen, The Netherlands) using a previously described protocol [10]. Oxygen uptake (AEI Metabolic System (Moxus) software, Pittsburgh, PA) and heart rate (Polar T31 Heart Rate Monitor; Polar Electro Inc., Woodbury, NY, USA) were sampled at 30-second epochs and rating of perceived exertion (RPE) using Borg's 0–10 scale [11] was assessed every minute to gauge subjective perception of physical effort both centrally (heart and breathing) and peripherally (arms).

Following peak arm ergometry testing, participants were strapped into the robotic limbs of the Lokomat and completed maximal lower limb isometric strength measures of hip and knee flexion/extension using the L-FORCE v2.0 software module (Lokomat System V5.0). The L-FORCE v2.0 has been technically and clinically validated [12].

#### 2.1.2. Body Weight Supported Walking Sessions

The subsequent 3 body weight supported walking sessions all used the same amount of support that had been established at the first session using the ZeroG (see* Baseline Assessments*). Participants completed walking trials in a* randomized* order using the Andago GmbH treadmill system (with or without the Lokomat) or overground ZeroG. EMG electrodes (Delsys Trigno Wireless EMG Surface Electrodes, Delsys, Boston, MA) were placed on tibialis anterior (TA), rectus femoris (RF), biceps femoris (BF), and medial gastrocnemius (MG) muscles of both legs. Footswitches (Delsys Wireless Force Sensitive Resistors, Delsys, Boston, MA) were placed on the bottom of both feet (sensor on base of first toe and calcaneus, resp.) to synchronize the muscle activation signals with heel strike as the subject walked. The subjects put on a Polar HR Monitor and then were fitted to the appropriate harness and device settings based on the prescreening evaluation and randomization order. Then the participant was attached to the portable metabolic cart (MedGraphics V02000, Medical Graphics Corp., St. Paul, MN) using the patented preVent mask. Resting measures (5 minutes) were taken before and after each walking session; oxygen uptake (sampled in 30-second epochs), HR, and brachial blood pressure were recorded at 1, 3, and 5 minutes. Participants walked at a comfortable self-selected speed at the same level of BWS regardless of the device until steady state exercise conditions (stabilized oxygen uptake and heart rate values; ±5 mL/kg/min and ±5 BPM, resp.) were achieved (~2 minutes). Participants were then asked to complete additional 2 minutes of steady state walking to allow for collection of oxygen uptake, HR, RPE, and muscle activity. Oxygen uptake and HR were recorded at 30-second epochs and RPE (central and peripheral; anchored to VO_2_ peak test) using Borg's 0–10 scale was monitored every minute. Muscle activity was continuously monitored throughout steady state walking sessions and collected using EMGworks Acquisition software program (Delsys EMGworks 4.0, Delsys, Boston, MA). For the Lokomat walking trial, all participants were asked to contribute as much as possible to the “walking motion”; the Guidance Force Control system was set to 100% for all sessions indicating full robotic assistance. When the robotic orthosis was not used, participants stepped on the treadmill* without* assistance and no corrections were made by assisting therapists to normalize stepping patterns. Participants were allowed to use the bilateral handrails on the treadmill to maintain postural stability, although they were asked to minimize upper-extremity weight bearing. Following all sessions, regardless of randomization order, participants were required to take a couple of steps using the ZeroG in order to have muscle activity data to normalize and control for potential day-to-day variation in EMG signal conduction and/or electrode placement. For all walking trials, able-bodied participants completed 2-minute steady state sessions (a) at baseline (0 BWS) and (b) at a percentage of BWS determined by their matched SCI participant (% BWS).

### 2.2. Data Analysis

MOXUS (AEI Metabolic System (Moxus) software, Pittsburgh, PA) and VO2000 (Medical Graphics Corp., St. Paul, MN) software programs were used for analysis of peak and walking VO_2_ metabolic measures, respectively. Oxygen consumption during walking sessions using the Lokomat, Manual Treadmill, and ZeroG devices was expressed relative to resting metabolic equivalents (METS), which are considered 2.7 mL/kg/min and 3.5 mL/kg/min for SCI and CON, respectively [13]. EMGworks Analysis (Delsys EMGworks 4.0, Delsys, Boston, MA) was used for analysis and filtering of muscle activity signals; the rectified EMG records were low-pass filtered using a second order Butterworth filter.

#### 2.2.1. Statistical Analysis

SPSS 17.0 (Chicago, IL) software program was used for statistical analysis of acquired data. The Shapiro-Wilk test was used to test for normality of the data due to the small sample size; nonparametric statistics were used where appropriate. Statistical significance was set at *P* < 0.05 for all analyses. Bonferroni's correction was used for post hoc testing when necessary. All reported values are expressed as mean ± standard error.

Repeated measures ANOVAs were conducted on the steady state cardiovascular data normalized to (a) resting (METS) and (b) VO_2_ peak. Repeated measures ANOVAs were conducted on the filtered root mean square (RMS) muscle activity signals over 3 consecutive gait cycles normalized to ZeroG filtered activity signals at the* same* level of BWS for each given day. For CON, this analysis was completed for both baseline (0 BWS) and matched (% BWS) conditions. Bivariate correlations (Pearson) were conducted for participants with SCI to investigate potential relationships between the amount of required BWS and flexion: extension isometric strength at the hip and knee, respectively. One-way ANOVAs were used to look at differences between SCI and CON.

## 3. Results

### 3.1. Baseline Assessments

**Figure 2:**
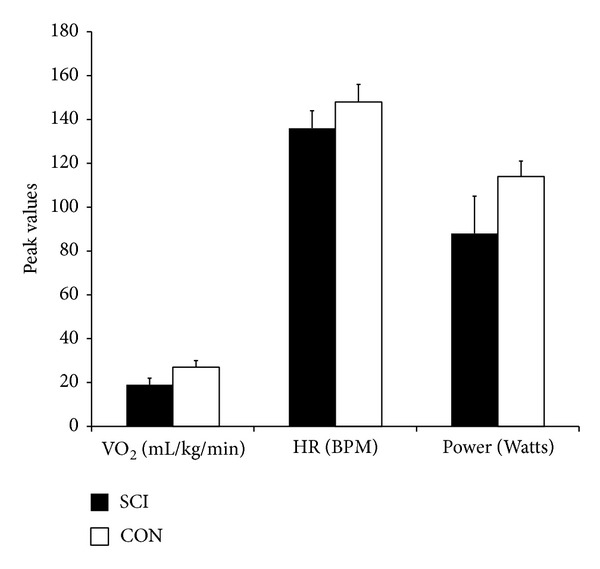
VO_2_ peak test variables for SCI and CON. Relative VO_2_ peak (mL/kg/min), peak heart rate (HR) expressed in beats per minute (BPM), and maximum power achieved (Watts). Values are mean ± SE.

**Figure 3:**
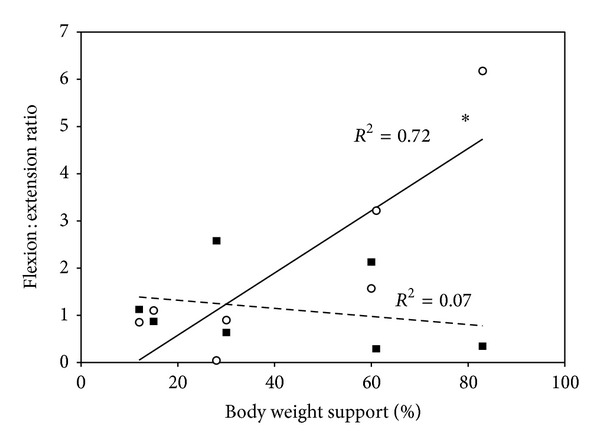
Relationship of the lower limb flexion: extension strength ratios (at the hip and knee) determined using LForce on the Lokomat with the percentage of body weight support (BWS) required for locomotion using the ZeroG in participants with SCI. Open circles and solid line indicate the relationship at the* hip*. Closed squares and dashed line indicate the relationship at the* knee*. *P* < 0.05 = ∗.

Seven individuals with incomplete SCI and seven gender-and-age matched controls participated in the study. No significant differences were found between SCI and CON with respect to demographic or aerobic peak measures from the arm ergometer test (Table 1, Figure 2). The average amount of BWS that was used for the walking sessions was 41.3% ± 10.16% (Table 1). A strong positive correlation (*R* ^2^ = 0.72) was found between the flexion: extension strength ratio at the hip in participants with SCI and the amount of BWS required to complete the overground walking session; the higher the flexion: extension ratio, the more support that was required (Figure 3). The greater contributor to this relationship at the hip joint was the decrease in isometric hip extension strength compared to flexion strength (*R* ^2^ = −0.69 versus *R* ^2^ = −0.36).

### 3.2. Body Weight Supported Walking Sessions

All 7 matched pairs completed the Manual Treadmill and ZeroG walking sessions; one participant in the SCI group did not complete the Lokomat walking session due to spasticity (exaggerated stretch reflexes). All participants reached steady state within 2 minutes of walking.

#### 3.2.1. Aerobic Demand Expressed Relative to Resting Values

**Figure 4:**
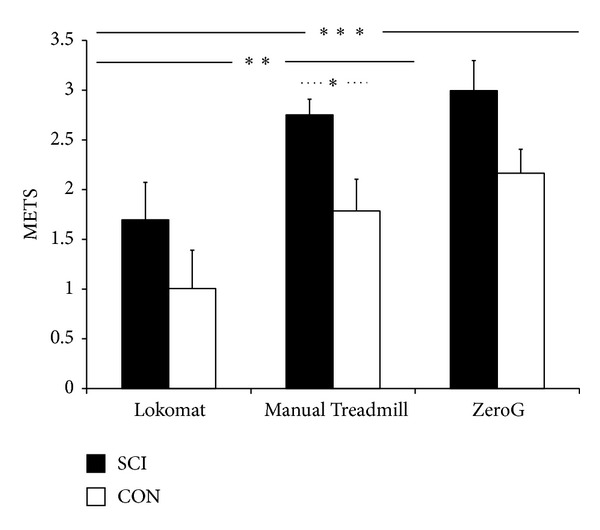
Metabolic equivalents (METS) for SCI and CON during locomotion with body weight support (BWS). Solid line indicates significance between* device* comparisons, and dashed line indicates significance between* group* comparisons. *P* < 0.05 = ∗, *P* < 0.01 = ∗∗, and *P* < 0.001 = ∗∗∗.

Lokomat sessions resulted in significantly lower MET values when compared to the Manual Treadmill or ZeroG sessions (Figure 4). The highest MET values were attained during ZeroG walking, although these were not statistically different compared to the Manual Treadmill, for both SCI and controls (3.0 ± 0.30 versus 2.8 ± 0.16 and 2.2 ± 0.24 versus 1.8 ± 0.32, resp.). Individuals with SCI achieved a significantly higher MET value compared to CON during the Manual Treadmill session (2.8 ± 0.16 versus 1.8 ± 0.32).

#### 3.2.2. Aerobic Demand Expressed Relative to Peak Values

**Figure 5:**
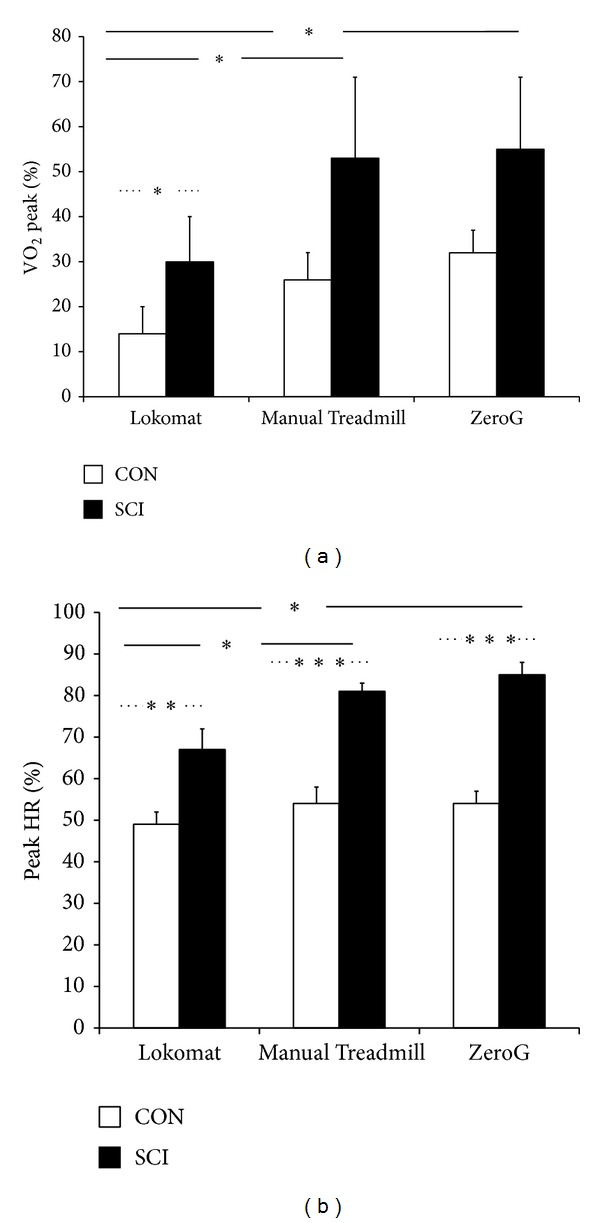
Cardiovascular measures ((a) VO_2_, (b) HR) during locomotion with body weight support (BWS) expressed as percentage of peak values obtained from the arm ergometer test. Solid line indicates significance between* device* comparisons, and dashed line indicates significance between* group* comparisons. *P* < 0.05 = ∗, *P* < 0.01 = ∗∗, and *P* < 0.001 = ∗∗∗.

Cardiovascular measures were expressed relative to percentage of peak values obtained during the arm ergometer test (Figure 5).

*VO*_2_. The Lokomat session was significantly less demanding compared to the Manual Treadmill and ZeroG sessions (e.g., 30.1% ± 10.06% versus 52.9% ± 17.60% versus 54.7% ± 16.42%, resp.) for participants with SCI (Figure 5(a)). Differences between groups existed during the Lokomat session only, with participants with SCI requiring a significantly greater percentage of peak VO_2_ compared to CON (30.1% ± 10.06% versus 14.4% ± 6.32%).

*HR.* Lokomat sessions resulted in significantly lower HRs (expressed as a percentage of peak) in comparison to the Manual Treadmill or ZeroG sessions (e.g., 67.3% ± 4.77% versus 80.8% ± 1.95% versus 84.7% ± 3.03%, resp.) for participants with SCI (Figure 5(b)). Individuals with SCI achieved a significantly higher percentage of peak HR values compared to CON during all 3 walking sessions (average of 77.7% ± 3.57% versus 52.3% ± 1.09% across trials).

*RPE.* Lokomat sessions were perceived to be significantly less demanding when compared to the Manual Treadmill or ZeroG sessions (e.g., central RPE: 0.5 ± 0.19 versus 3.7 ± 1.28 versus 3.8 ± 1.14; peripheral RPE: 0.7 ± 0.18 versus 4.1 ± 0.64 versus 5.1 ± 1.18) for participants with SCI. Additionally, individuals with SCI perceived the Manual Treadmill and ZeroG sessions to be significantly more demanding compared to CON (e.g., central RPE: 3.7 ± 1.28 versus 0.9 ± 0.14; 3.76 ± 1.14 versus 1.0 ± 0.00; peripheral RPE: 4.1 ± 0.64 versus 0.9 ± 0.86; 5.1 ± 1.18 versus 1.0 ± 0.00).

#### 3.2.3. Muscle Activity Expressed Relative to Walking While Using the ZeroG

**Figure 6:**
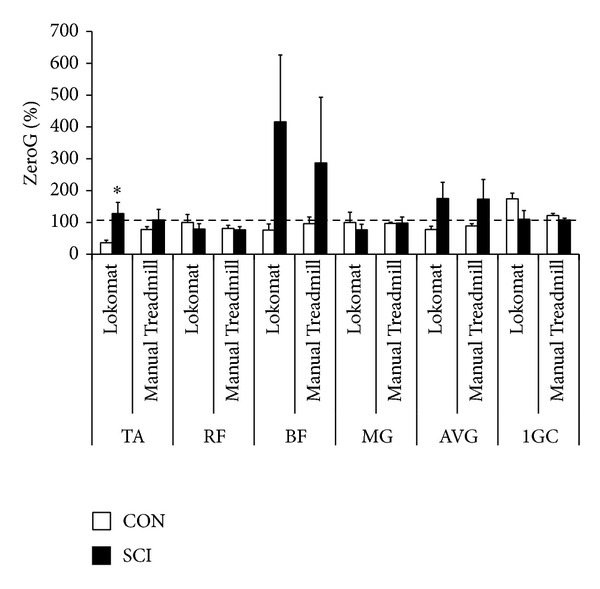
Muscle activity of independent muscle groups during treadmill locomotion with body weight support (BWS) normalized to ZeroG stepping with the same BWS. TA indicates tibialis anterior, RF indicates rectus femoris, BF indicates biceps femoris, MG indicates medial gastrocnemius, AVG indicates average muscle activity over a gait cycle, and 1GC indicates gait cycle completion time. Dashed line at 100 = value obtained while walking on the ZeroG. Statistically significant between group (SCI versus CON) comparisons *P* < 0.05 = ∗.

**Figure 7:**
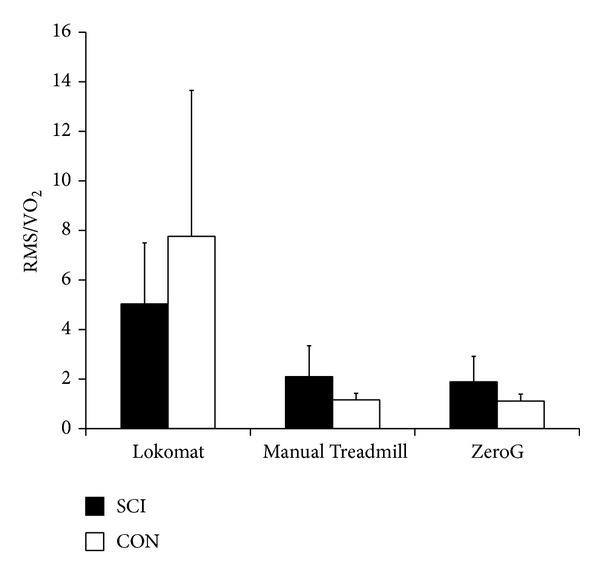
Ratio of root mean square (RMS) muscle activity across all muscle groups (*μ*V) to oxygen uptake (%VO_2_ peak) between devices for SCI and CON. Mean ± standard error.

Nonstatistically significant differences in muscle activity gait parameters were found between the three devices (Figures 6 and 7). For individuals with SCI, average muscle activation tended to be higher for both treadmill conditions compared to the ZeroG session, which could be attributed to increases in TA and BF activity. Conversely, the ZeroG session tended to require greater muscle activation compared to the treadmill sessions for CON. The only statistically significant difference between groups occurred during the Lokomat session, which elicited greater relative TA activation for participants with SCI compared to CON (128.3% ± 35.07% versus 36.0% ± 8.22%).

## 4. Discussion

The main objective of this study was to compare physiological responses during 2 minutes of steady state locomotion at the* same* level of BWS while using the Lokomat, Manual Treadmill, and ZeroG. It was hypothesized that ZeroG locomotion would be the most physiologically demanding session for individuals with incomplete SCI as it most resembles unsupported overground walking. As expected walking sessions were physiologically more demanding for individuals with SCI compared to CON. Contrary to what was hypothesized, both the Manual Treadmill and ZeroG sessions were considered significantly more demanding compared to the Lokomat, with no significant differences between the two sessions.

### 4.1. Baseline Assessments

VO_2_ peak values obtained from the men with incomplete SCI (1.7 ± 0.27 L/min) were compared to normative physical capacity values. There was no difference in VO_2_ peak between individuals with paraplegia or tetraplegia whose capacities were fair and excellent, respectively, based on the literature [14]. Peak VO_2_ values from able-bodied participants (2.2 ± 0.15 L/min) were similar to those reported by van Loan and colleagues [15] (2.1 L/min) and more recent unpublished data from our lab (2.4 L/min). Interestingly, the peak aerobic data in this study was similar between the two groups, which are contrary to other studies indicating that those with SCI usually obtain lower values [16] due to a decreased amount of active muscle mass and sympathetic tone limiting venous “muscle pumping” action and the ability to increase oxygen uptake [17]. Zwiren and Bar-Or [18], however, found no significant differences in VO_2_ max in matched wheelchair-active and normal active subjects, suggesting that conditioning levels of the participants in the current study may have been more similar than typically expected (e.g., two participants with SCI were competitive athletes).

We used the L-Force module in the Lokomat to assess lower extremity isometric strength of the hip and knee. The reduced hip extension and knee flexion strength measures obtained from participants with SCI compared to CON are in agreement with previous studies which suggest that this population has difficulty voluntarily activating muscle below the lesion level making weight bearing and toe-clearance during gait difficult [19]. Further investigation into the flexion: extension strength ratio at both the hip and knee determined that a positive correlation existed between flexion: extension strength at the hip (primarily due to a reduction in hip extensor isometric strength) and the percentage of BWS required to complete an overground walking session using the ZeroG. The importance of the hip extensors for locomotion is in agreement with a study by Yang and colleagues [20], who found that manual muscle testing scores for the hamstrings in addition to the quadriceps were the strongest predictors of responsiveness to body weight supported gait training. In fact, responders on average had twice the volitional muscle strength as that of nonresponders. Whether the isometric strength measures obtained from the L-Force module of the Lokomat are able to predict responsiveness to body weight supported gait training, however, requires further investigation. This may provide a useful tool to therapists in terms of exercise prescription, determining readiness for locomotor training, as well as monitoring rehabilitation progression.

### 4.2. Randomized Body Weight Supported Sessions

#### 4.2.1. Body Weight Support

The average amount of BWS used during the walking sessions was 41.3%, with only three of the seven participants with SCI able to complete walking sessions with the recommended less than 30% BWS [1]. These lower levels of support have been shown to better resemble independent overground walking patterns while allowing individuals to better maintain upright posture.

#### 4.2.2. Metabolic Demand

Previous research has indicated the robotic orthosis results in lower metabolic costs (approximately 20%) compared to the Manual Treadmill [21], with the potential to minimize these differences if the participant is encouraged to exert maximal effort [9]. In this study, despite encouraging participants to maximally contribute to the walking motion in all walking conditions differences in metabolic costs were evident, with the Lokomat resulting in the lowest metabolic demand (approximately 23.8% of VO_2_ peak for the SCI group) compared to the Manual Treadmill and ZeroG sessions, which had similar oxygen costs. While this may suggest that perhaps participants were not providing maximal efforts during these sessions, it is also important to note that in order to standardize walking conditions between participants 100% Guidance Force was set on the robotic orthosis which may have limited the ability for our participants to exert a maximal effort. Further, the study by Israel and colleagues [9] provided therapist assistance during treadmill sessions, an option that was not provided for participants in this study. Other features of the Lokomat that could have contributed to the differences in metabolic costs include posterior support assisting with forward propulsion and stability at the pelvis and trunk. The evidence from this study suggests that the use of the robotic orthosis is not entirely passive, with increases in oxygen uptake evident during training sessions, which according to Krewer and colleagues [22] can be attributed to loading during stance phase resulting in associated muscle activation.

In order to complete a walking session using the overground device at the same level of BWS, the participants with SCI required three times resting metabolic rate while their matched controls only required double. The walking sessions completed with the ZeroG and Manual Treadmill sessions required greater than 50% of VO_2_ peak values for the SCI group (e.g., 55% and 53% of VO_2_ peak, resp.). This would suggest that walking using the aforementioned devices may be above the anaerobic threshold [23, 24] for people with SCI, resulting in reduced endurance and earlier onset of fatigue. In contrast, the ZeroG and Manual Treadmill sessions required only 32% and 26% of peak oxygen uptake for the CON group. This is consistent with evidence obtained by Waters and colleagues [25] who found that individuals without mobility impairments require minimal effort during walking with rates of oxygen consumption of 30% of maximum aerobic capacity. The similarity in oxygen costs between the treadmill session without the robotic orthoses and the overground walking session in CON is in agreement with evidence in able-bodied populations which suggest that no significant differences exist in energy expenditure between treadmill and overground walking at controlled velocities [23]. It is important to note that walking velocity in this study was not controlled, although all participants received the same instruction to walk at a comfortable speed. Oxygen uptake while walking overground in this study was 41.8% higher in SCI compared to noninjured adults, which is similar to earlier work which found the rate of oxygen uptake while walking to be 38% higher in SCI compared to CON [26]. In the present study individuals with incomplete SCI had 36.5% higher HR compared to CON during overground walking sessions which is slightly greater than the 24% greater increase found during walking by Teixeira da Cunha-Filho and colleagues [27].

#### 4.2.3. Muscle Activity

Muscle activity for SCI tended to be greater with the treadmill than the ZeroG due to increases in TA and BF activity. For the SCI group, the “foot lifters” used with the Lokomat may have provided afferent feedback to the spinal cord during gait encouraging dorsiflexion. Evidence from this study supports this as Lokomat sessions resulted in greater TA activation in SCI compared to CON. While the “foot lifters” used with the robotic orthosis may have provided beneficial feedback for the SCI group, reduced EMG activity of TA during gait in the CON group may have occurred using this same device as the foot lifters may have inhibited normal activation of this muscle group.

It has been suggested that muscles with greater cortical projections such as TA and more proximal muscles such as the hamstrings [28] are the most affected in individuals with SCI following body weight supported training [29]. Improved hip extension with training is common with treadmill training as the belt encourages hip extension forcing individuals to “pull up” (e.g., increasing knee flexion) during swing. The large variability in BF activation in this study may be an indicator of potential responders to body weight supported training. Gorassini and colleagues [7] showed increased TA and hamstring muscle activation during treadmill walking only in responders. While responders increased amplitude of hamstring activity, burst duration decreased resulting in less cocontraction with quadriceps activation. Thus, the ability to modify muscle activation patterns after SCI may predict responsiveness to training.

Unlike the SCI group, muscle activity for CON tended to be greater with overground sessions rather than treadmill sessions, as well as having higher TA activation during supported Manual Treadmill versus Lokomat sessions. Muscular work associated with forward propulsion is thought to be a primary determinant of the metabolic costs of walking in subjects without neurological injury [9] which would be greater when using the ZeroG compared to the treadmill sessions, especially when the robotic orthosis is used. As previously mentioned, “foot lifters” used with the robotic orthosis may have inhibited normal activation of TA for the CON group, resulting in the obtained differences between the two treadmill modalities.

While both groups were able to successfully complete all walking sessions greater muscle activity was evident during treadmill sessions in the SCI group compared to during ZeroG sessions for the CON group. This may provide evidence in support of the idea of motor equivalence [30]. Essentially this principle suggests that a given motor task goal (e.g., walking) can be achieved using different muscle synergies. This is advantageous for individuals with lesions to the spinal cord as they can take advantage of the redundancies of the neuromuscular system to accomplish motor tasks. For example, individuals with SCI may require use of their arms and/or axial muscles for support during swing phase, essentially completing the same phase of gait but at a greater energy cost due to the involvement of additional musculature. As an individual progresses with training, neuronal coupling of movement (e.g., incorporation of arm swing) will provide additional sensory feedback to help generate temporally appropriate muscle activity patterns [31]. From a therapeutic perspective, therefore, body weight supported training interventions help individuals to learn to produce new motor strategies in a controlled setting with the hopes of transferring this rehearsed pattern to unsupported overground walking.

### 4.3. Limitations

The number of participants in this study was arguably small; however, the inclusion criteria of having the ability to take independent steps using the ZeroG limited the available sample pool. Therefore, the generalizability of these findings may be applicable only to individuals with higher levels of motor function following an incomplete SCI. It has been demonstrated in the literature that gait kinematics similar to baseline walking (0% BWS) can be maintained when 30 percent or less support is provided [5]. Recent work from our lab, specifically looking at changes in muscle activation with increased amounts of BWS using the ZeroG in able-bodied adults, found significant reductions in muscle activity when 40 or more percent of body weight was offset by the device without altering the muscle activation pattern during gait [31]. It is important to note that 3 of the 7 participants with incomplete SCI would have been unable to independently complete the walking sessions if <40% BWS was provided on the ZeroG. Whether these individuals can obtain the recommended ranges of support with training is an area of future investigation and will help determine if the comparison between devices is minimized at lower levels of support.

The Lokomat was programmed to provide maximal assistance to the legs during all phases of gait (e.g., 100% Guidance Force) in order to standardize the Guidance Force, therefore these results are only relevant to Lokomat sessions completed under these conditions. It is anticipated that decreasing the Guidance Force will increase physiological demand; however, whether these changes result in statistically nonsignificant differences between the two treadmill conditions has yet to be determined.

Participants were allowed to use the treadmill handrails or the arms of the therapist during the overground session for balance but were discouraged to use them for weight bearing. Without the use of arm swing, individuals were unable to take advantage of neuronal coupling, which may have influenced lower extremity muscle activity [32]. While attempts were made to ensure consistent upper-extremity use across stepping conditions no objective measures of force (e.g., force plates on handrails) were made to ensure this.

Finally, the inability to control walking speed, particularly with respect to the overground walking session, may have influenced the results obtained, as reduced speed has been associated with increased signal variability and decreased muscle activation. According to Winter [33], changes in walking speed affect the acceleration of lower limbs during gait, primarily activity at the hip and knee versus the ankle. Therefore, while our EMG recordings may have been differentially affected by not having control over this factor (e.g., RF and BF greater than TA and MG), it would have been close to impossible to control walking speed during the ZeroG sessions.

## 5. Conclusions

The objective of this study was to compare oxygen demand and muscle activation between the Lokomat, Manual Treadmill, and ZeroG. Consistent with our hypothesis, walking sessions were more demanding for participants with SCI compared to CON. Contrary to our hypothesis the ZeroG was not considered the most physiologically demanding session, with both the ZeroG and Manual Treadmill sessions eliciting significantly greater VO_2_ values compared to the Lokomat. In addition, contrary to what was expected, the ZeroG did not elicit significantly greater lower limb muscle activity in the four muscle groups included in this investigation. The evidence from this study would suggest a benefit to using the Lokomat to work on isolated hip extension strength. Therapists can take advantage of the feedback system of the device, the greater BF activation of this treadmill-based exercise, as well as having the ability to conduct longer sessions due to the decreased cardiovascular and muscular demands imposed on the patient using the robotic device. The Manual Treadmill and ZeroG should then be used as more intense progressions where hip extension can continue to be encouraged while using the treadmill and additional components of gait (e.g., balance and torso stability) can be focused on while using the overground device.

## Figures and Tables

**Table 1:** Demographic variables for SCI and CON.

	SCI		CON		Between groups (*P*-value)
	Mean ± Standard error	Range	Mean ± Standard error	Range	
Age (years)	42.6 ± 4.29	23–55	42.7 ± 5.40	20–58	0.98
Height (cm)	179.1 ± 1.56	173–184	177.5 ± 2.91	168–190	0.84
Weight (kg)	89.6 ± 6.39	81.2–107.6	84.8 ± 5.88	69.4–112.1	0.52
BMI (kg/m^2^)	27.9 ± 2.03	20.6–36	26.1 ± 1.46	20.6–31.1	0.48
BWS (kg)	37.8 ± 9.62	10–68	32.8 ± 7.40	10–68	0.67
BWS (%)	41.3 ± 10.16	12–83	41.3 ± 10.16	12–83	1.00
Time since injury (years)	4.0 ± 0.62	2–7	N/A		N/A

SCI: incomplete spinal cord injury; CON: matched controls; BMI: body mass index; BWS: body weight support.

## Abbreviations

ADL: Activities of daily living
ANOVA: Analysis of variance
AIS: American Spinal Injury Association Impairment Scale
BF: Biceps femoris
BMI: Body mass index
BPM: Beats per minute
BWS: Body weight support
CON: Able-bodied controls
EMG: Electromyography
HR: Heart rate
MET: Metabolic equivalent
MG: Medial gastrocnemius
RF: Rectus femoris
RMS: Root mean square
ROM: Range of motion
RPE: Rating of perceived exertion
SCI: Spinal cord injury
TA: Tibialis anterior
VO_2_: Metabolic rate of oxygen consumption.
